# Blood Sampling From Rat Ileal Mesenteric Vein Revealed a Major Role of Dietary Protein in Meal-Induced GLP-1 Response

**DOI:** 10.3389/fendo.2021.689685

**Published:** 2021-06-02

**Authors:** Tohru Hira, Madoka Sekishita, Hiroshi Hara

**Affiliations:** ^1^ Research Faculty of Agriculture, Hokkaido University, Sapporo, Japan; ^2^ Graduate School of Agriculture, Hokkaido University, Sapporo, Japan; ^3^ Faculty of Human Life Science, Fuji Women’s University, Ishikari, Japan

**Keywords:** postprandial GLP-1 response, dietary protein, ileal mesenteric vein, portal vein, gut hormone

## Abstract

The present study was conducted to examine region-dependent glucagon-like peptide-1 (GLP-1) responses to “meal ingestion” under physiological (conscious and unrestrained) conditions using rats with a catheter inserted into either the portal vein (PV) or the ileal mesenteric vein (ILMV). After recovery from the cannulation surgery, blood samples were collected from either PV or ILMV catheter before and after the voluntary ingestion of test diets. After an AIN-93G standard diet ingestion, GLP-1 concentration was higher in ILMV than in PV, and postprandial responses of peptide-YY (PYY) had similar trend, while that of glucose dependent-insulinotropic polypeptide showed an opposite trend to GLP-1/PYY responses. In a separated experiment, a protein-enriched diet containing casein at 25% wt/wt transiently increased GLP-1 concentration only in ILMV; however, a protein-free diet did not increase GLP-1 concentrations in PV or ILMV. These results indicate that postprandial GLP-1 is immediately released from the distal intestine under physiological conditions, and that dietary protein has a critical role in the enhancement of postprandial GLP-1 response.

## Introduction

Gut hormones are considered to be locally produced in specific regions of the gastrointestinal tract (1, 2). Thereby, it has been also considered that secretin, cholecystokinin (CCK), and glucose dependent-insulinotropic polypeptide (GIP) are immediately released from the proximal small intestine, while glucagon like-peptide-1 (GLP-1), glucagon like-peptide-2 (GLP-2), and peptide-YY (PYY) are released from the distal intestine later, in response to meal ingestion. However, GLP-1 and PYY production in the proximal small intestine (3–5) could contribute to a rapid response of these hormones (6, 7) after a meal. There is no study to prove this hypothesis under conscious conditions in an animal model.

The primary purpose of the present study was to demonstrate regional gut hormone responses to voluntary meal ingestion under physiological (conscious and unrestrained) conditions, in a rat model. For this purpose, we developed surgical model rats by inserting a catheter into the mesenteric vein of the distal small intestine (ileal mesenteric vein, ILMV). We firstly compared gut hormone concentrations in the plasma samples collected from the ILMV to those from the portal vein (PV) of another group of rats with a portal catheter (8, 9).

In our previous studies using rats, oral administration of dietary peptides (2 g/kg dose) significantly increased plasma GLP-1 concentrations (10, 11), while the same dose of glucose did not (10, 12). These observations raised a hypothesis that proteins in the meal have a critical role in postprandial GLP-1 secretion. To test this hypothesis, we further examined the role of dietary protein in postprandial GLP-1 responses using the PV- or ILMV-cannulated rats, under a voluntary feeding condition.

## Materials and Methods

### Animals and Diets

Male Sprague–Dawley rats (7 weeks old) were purchased from Japan SLC (Hamamatsu, Japan). All the animals were housed in individual cages and had free access to water and a semi-purified AIN-93G diet (13). All animal experiments were performed after an acclimation period (3−7 days) in a temperature-controlled room maintained at 23°C ± 2°C with a 12-h light/dark cycle (08:00−20:00 h, light period). This study has been approved by the Hokkaido University Animal Committee, and animals were maintained in accordance with the Guide for the Care and Use of Laboratory Animals of Hokkaido University.

### Surgical Preparation

**Figure 1:**
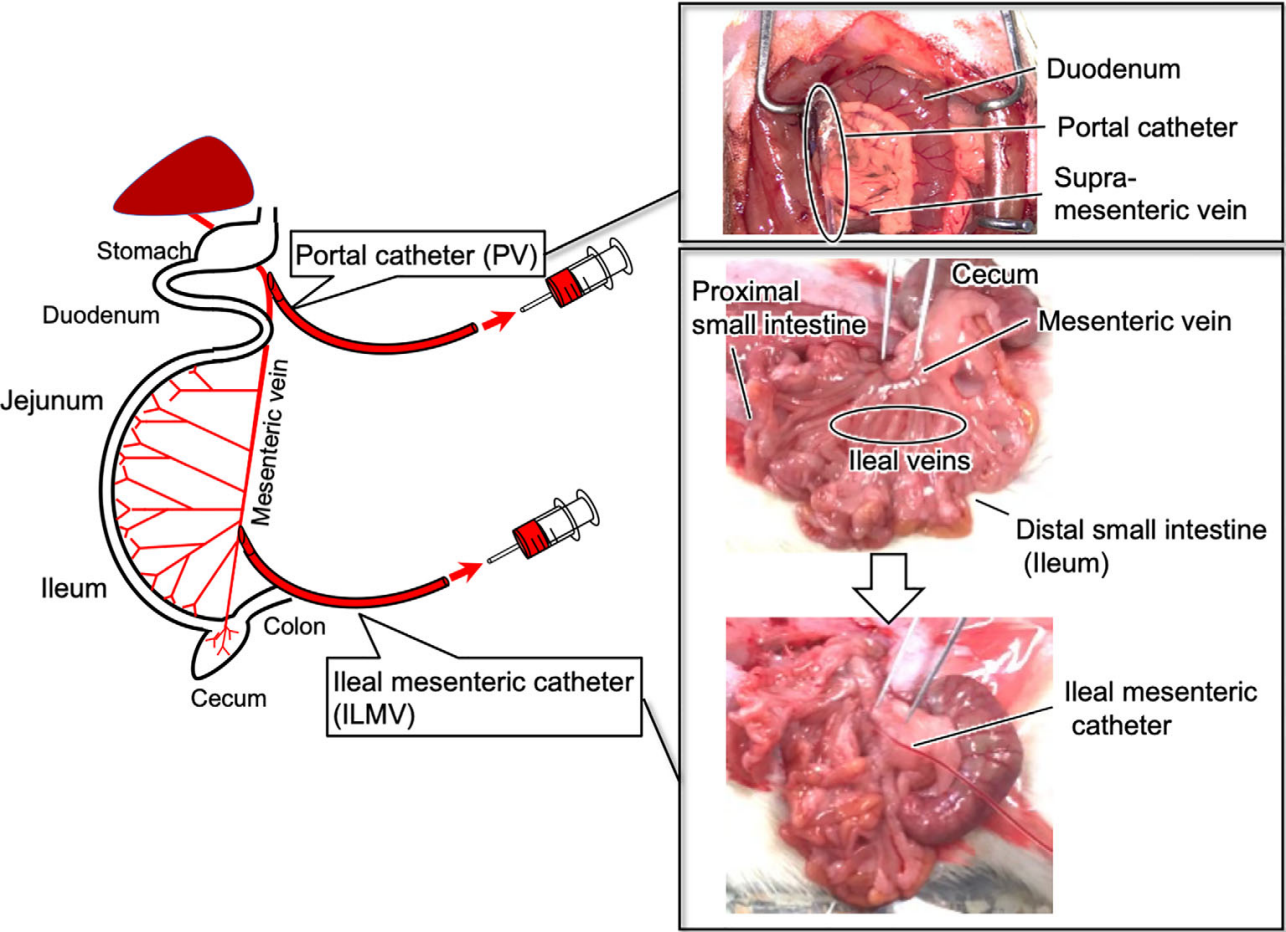
Summary of cannulation into the ileal mesenteric vein and the portal vein in rats.

Rats were anesthetized with sodium pentobarbital (50 mg/kg body weight; Somnopentyl Injection, Kyoritsu Seiyaku Co., Tokyo, Japan), and then the laparotomy was performed. The small tip (6–7 mm) of a polyethylene catheter (SP 28; ID 0.4 mm, OD 0.8 mm for PV, SP 10; ID 0.28 mm, OD 0.61 mm for ILMV, Natsume Seisakusyo, Tokyo, Japan) was connected to a silicone catheter (Silascon no. 00, ID 0.5 mm, OD 1.0 mm; Dow Corning Co.) approximately 30 cm long. The polyethylene catheter was directly inserted into the PV and/or ILMV at the position where 2-3 of ileal veins join (**Figure 1**), and then fixed with an instant adhesive. The catheter was prefilled with sterile saline that contained heparin (50 IU/ml; Ajinomoto, Tokyo, Japan). The free end of the catheter was dorsally exteriorized and fixed behind the neck, and the abdomen was then closed with surgical threads. The abdominal skin incision was closed with Michel clips. We flushed the catheter with heparinized saline daily to maintain patency. After a recovery period of 3–4 days, rats without catheter-clogging were used for following experiments. Using these model rats, we conducted the experiments under non-anesthetized and unrestrained conditions (8). After the experiments, rats were euthanized by exsanguination under anesthesia with sodium pentobarbital (50 mg/kg body weight).

### Meal Tolerance Tests (MTTs)

During the acclimation period, rats were trained to consume a certain amount of diet (10 g/kg) within 60 min, after overnight fasting. After the recovery period, rats were fasted for 24 h, and the basal (0 min) blood sample (200 µL), was collected from the catheter. The rats were then provided the test diet (10 g/kg, **Table 1**) for 60 min. Blood samples (200 µL each) were collected at 15, 30, 60, 90, and 120 min after providing the diet. MTTs were started around 10:00 AM and conducted during the light period. All blood samples were mixed with ethylenediamine tetraaceticacid (EDTA) solution (1.8 mg/mL blood), Pefabloc SC (AEBSF; 4-(2-aminoethyl)benzenesulfonyl fluoride; Roche, 1 mg/mL blood), Protease inhibitor Cocktail (P2714; SIGMA, 1 µL/mL blood), and DPP-IV inhibitor (DPP4-010; Millipore, 10 µL/mL blood), in a syringe. Plasma was separated and frozen at −80°C until measurement of glucose and gut hormone concentrations. Plasma glucose concentrations were measured using the Glucose CII test kit (Wako). Active GLP-1, total GIP, total PYY, and insulin concentrations were measured using the MILLIPLEX MAP Rat Metabolic Hormone Magnetic Bead Panel (Millipore, Billerica, MA). Intra-assay precision is 1–8%; inter-assay precision is 7–29%; standard curve ranges for GLP-1 (Active) is 41–30,000 pg/mL, for GIP (Total) is 2.7–2,000 pg/mL, for PYY (Total) is 7–5,000 pg/mL, respectively. Total GLP-1 levels were measured using the ELISA kits (EZGLP1T-36K, Millipore). Intra-assay precision is < 5%; inter-assay precision is < 12%; standard curve range is 4.1–1000 pM.

**Table 1:** Compositions of diets.

Ingredient	Experiment 1		Experiment 2	
	AIN-93G		Control	Protein-free
			g/kg of diet	
Casein^1^	200		250	0
Cornstarch	397.486		350.486	600.486
Dextrinized cornstarch^2^	132		132	132
Sucrose	85		70	70
Soybean oil	70		70	70
Cellulose^3^	65		50	50
Mineral mixture^4^	35		35	35
Vitamin mixture^4^	10		10	10
L-Cystine	3		0	0
Choline bitartrate	2.5		2.5	2.5
*tert*-Butylhydroquinone	0.014		0.014	0.014
Energy density (kcal/g)	3.9		3.9	3.9

^1^Acid Casein (Fonterra, Ltd., Auckland, New Zealand).

^2^TK-16 (Matsutani Chemical Industry Co., Ltd., Hyogo, Japan).

^3^JustFiber (Morimura Bros., Inc. Tokyo, Japan).

^4^Mineral and vitamin mixtures were prepared according to the AIN-93G formulation.

Experiment 1 was conducted to compare gut hormone and glycemic responses after ingestion of a standard meal. Rats with the PV or ILMV catheter were fed the standard diet (AIN-93G) containing casein at 20% as a protein source (**Table 1**).

Experiment 2 was conducted to compare gut hormone and glycemic responses to the diet with or without protein source (**Table 1**). The control diet contained casein (25% wt/wt) as a protein-enriched diet, in order to emphasize the impact of dietary proteins. The protein-free diet was devoid of casein, and the amount of casein (250 g/kg diet) was replaced by corn starch.

### Statistical Analysis

Results are expressed as mean ± SEM. Area under the curve (AUC) of plasma parameter concentrations during the MTT was calculated by the trapezoidal rule. Statistical significance was determined using two-way repeated-measures ANOVA to assess the main effects (time, region of blood sampling, or diet), as well as their interaction effects, using JMP Pro software version 12 (SAS Institute, NC, USA). Statistical significance (P < 0.05) between mean values was evaluated using Student’s t-test or Dunnett’s test as described in figure legends.

## Results

In experiment 1, rats consumed more than 95% of the diet (2.80 ± 0.05 g in the PV group and 2.75 ± 0.04 g in the ILMV group) provided at the dose of 10 g/kg, within 15 min. As both groups consumed almost equivalent amount of the diet, the following results could not be attributed to a difference in the amount of food intake.

**Figure 2:**
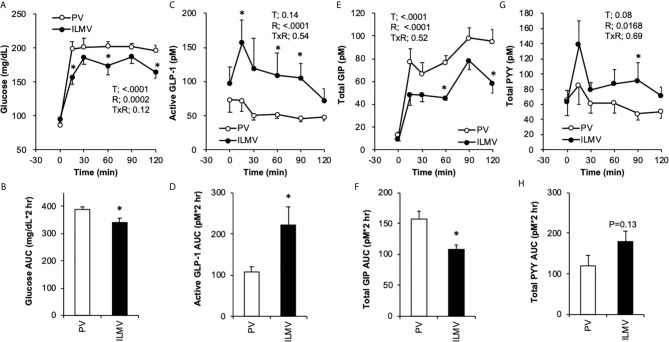
Changes in glucose and gut hormone concentrations in the portal vein (PV) plasma and in the ileal mesenteric vein (ILMV) plasma after ingestion of the standard diet. Rats were provided the AIN-93G diet at the dose of 10 g/kg body weight, after 24-h fasting. Blood samples were collected from the catheter before (0 min) and after providing the meal. Data are presented as mean values and SEM (n = 10 in PV, and n = 6 in ILMV). P values calculated by two-way repeated-measures ANOVA for effects of time (T), the region of blood sampling (R), and their interactions (TxR) are presented in each of the panels. Asterisks (*) indicate significant difference between the value at the same time point **(A, C, E, G)** or between the AUC **(B, D, F, H)** (P < 0.05, Student’s t-test).

Plasma glucose concentrations (**Figure 2A**) elevated immediately in both groups, glucose concentrations at 15, 60, and 120 min were significantly higher in the PV group than those in the ILMV group, accordingly, the PM group had higher AUC of glucose than the ILMV group (**Figure 2B**). The concentration of active GLP-1 increased in the ILMV group immediately (15 min) after ingesting the meal, whereas it showed no increment in the PV group (**Figure 2C**). The AUC of GLP-1 was higher in the ILMV group than in the PV group (**Figure 2D**). GIP concentrations continuously increased over time in both groups, but the increment and the AUC were higher in the PV than in the ILMV group (**Figures 2E, F**). The concentration of PYY transiently increased only in the ILMV group, and a significant difference between the regions was detected by two-way ANOVA (**Figure 2G**). The AUC of PYY was not significantly different between two groups (**Figure 2H**). Insulin concentrations were similarly elevated both in the PV and the ILMV, and not significantly different between the regions (**Supplementary Figures 1A, B**).

In experiment 2, rats with the PV catheter consumed more than 95% of test diets (2.63 ± 0.05 g in the control group, 2.51 ± 0.05 g in the protein-free group) provided at a dose of 10 g/kg, within 15 min. Rats with the ILMV catheter consumed more than 92% of test diets (2.48 ± 0.05 g in control group, 2.38 ± 0.11 g in the protein-free group) within 15 min, and more than 97% of the respective diets within 60 min.

**Figure 3:**
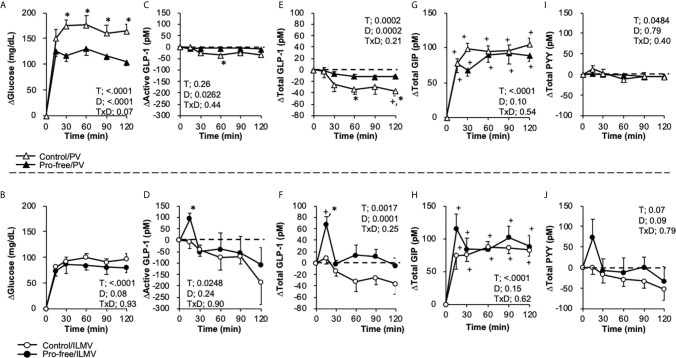
Changes in glucose, active and total GLP-1, and PYY concentrations in the portal vein (PV) plasma and the ileal mesenteric vein (ILMV) plasma after the ingestion of the diet-containing protein (control) or the diet-not containing protein (pro-free), respectively. Rats were provided the control diet (containing casein at 25% wt/wt) or the pro-free diet at the dose of 10 g/kg body weight, after 24-h fasting. Blood samples were collected from the catheters before (0 min) and after providing the meal. Results are presented as the change (Δ) from the basal values of individual parameters in each group. Data **(A, B)** glucose, **(C, D)** active GLP-1, **(E, F)** Total GLP-1, **(G, H)** Total GIP, **(I, J)** Total PYY) are presented as mean values and SEM (n = 10 in Control/PV, n = 8 in Pro-free/PV, n = 5 in Control/ILMV, n = 6 in Pro-free/ILMV for glucose, active GLP-1 and total PYY; n = 10 in Control/PV, n = 8 in Pro-free/PV, n = 4 in Control/ILMV, n = 5 in Pro-free/ILMV for total GLP-1). P values calculated by two-way repeated-measures ANOVA for effects of time (T), diet (D), and their interactions (TxD) are presented in each of the panels. Asterisks (*) indicate significant difference between the value at the same time point (P < 0.05, Student’s t-test). Plus (+) signs indicate significant difference compared to the basal value of each group (P < 0.05, Dunnett’s test). Dashed lines represent the basal level (Y = 0) in each of the parameters.

Basal (0 min) glucose concentrations (mg/dL) were similar in all the groups; 86.5 ± 2.5, 84.5 ± 4.0, 83.3 ± 4.4, and 90.9 ± 4.8 in Control/PV, Protein-free/PV (Pro-free/PV), Control/ILMV, and Protein-free/ILMV (Pro-free/ILMV), respectively. Glycemic response to both of test diets were higher in the PV group than in the ILMV group (**Figures 3A, B**).

Because basal values of gut hormone concentrations had unexpectedly high variances, results are presented as changes from the basal values (**Figure 3**). Active GLP-1 concentrations (pM) were 28.4 ± 7.1, 60.3 ± 17.3, 239.0 ± 146.1, and 292.1 ± 150.5; total GLP-1 concentrations (pM) were 25.7 ± 4.8, 54.8 ± 11.4, 46.8 ± 17.5, and 75.2 ± 27.0; total GIP concentrations (pM) were 9.6 ± 1.3, 8.8 ± 0.8, 13.9 ± 1.6, and 15.4 ± 2.5; PYY concentrations (pM) were 28.0 ± 6.0, 44.3 ± 6.6, 94.8 ± 55.8, and 106.1 ± 44.2, in the Control/PV, Pro-free/PV, Control/ILMV, and Pro-free/ILMV groups, respectively.

In the PV, plasma glucose was higher in the Pro-free group than the control group (**Figure 3A**). No significant differences were observed between two groups in the ILMV (**Figure 3B**). Active GLP-1 concentration was transiently (15 min) elevated only in the Control/ILMV group with a significant difference as compared to the value at the same time point in the Pro-free/ILMV group (**Figures 3C, D**). We additionally measured the total GLP-1 concentration, as it reflects GLP-1 release rather than biologically active GLP-1. Because plasma samples in two rats (one in the Control/PV group and another in the Pro-free/ILMV group) did not remain sufficient for total GLP-1 measurement, the data were obtained respectively from 4 and 5 rats in these groups. Only in the ILMV group, total GLP-1 concentration sharply increased (P < 0.05 vs 0 min within the group, and, vs Pro-free group at the same time point) 15 min after ingestion of the control diet (**Figures 3E, F**). The Protein-free diet failed to increase total GLP-1 concentrations in the ILMV group. Total GIP concentrations increased similarly by both of diets in the PV and ILMV, respectively (**Figures 3G, H**). The Protein-free diet failed to increase total GLP-1 concentrations in the ILMV group. Postprandial responses of PYY (**Figures 3I, J**) were overall similar to those of GLP-1 both in the PV and ILMV groups. Incremental AUC (**Supplemental Figure 2**) of glucose was higher in PV of the Pro-free group than in PV of the control group, and incremental AUC of total GLP-1was higher in ILMV of the control group than the Pro-free group.

## Discussion

It has been widely accepted that postprandial glucose absorption occurs in the proximal small intestine based on old and valuable studies (14–16). In the present study, differences in glycemia between the PV and ILMV groups probably reflects avid glucose absorption in the proximal small intestine. This is also supported by the result in experiment 2, in which the glycemic response was higher to the Pro-free diet than to the control diet in the PV group, but the difference was not observed in the ILMV group. To the best of our knowledge, this is possibly the first study demonstrating postprandial glycemia in veins of different regions of the intestine under physiological (conscious, unrestrained, and voluntary feeding) conditions.

Although we had expected similar profiles of GLP-1 responses, possibly at different degrees between the PV and ILMV groups, GLP-1 responses to the normal diet apparently differed. These results strongly suggest that GLP-1 is initially released from the ileum in response to meal ingestion. Such an interpretation agrees with the abundant distribution of GLP-1 producing cells in the distal than in the proximal small intestine (3–5). The immediate response (at 15 min) appears discrepant when considering the transit of luminal nutrients after meal ingestion. This could be explained by a “proximal-distal enteroendocrine loop” (17–19), in which certain proximal signals and the vagus nerve mediate GLP-1 release from the distal intestine. However, it is still possible that some of nutritional components immediately reached the middle-distal part of small intestine so that they directly stimulated L cells locally.

In contrast to GLP-1, postprandial GIP concentrations were higher in the PV than in the ILMV, which could be explained by the localization of GIP-producing K cells in the proximal small intestine (3–5). Such region-dependent responses in glucose, GLP-1, and GIP levels help to validate that our cannulation models (especially ILMV) are useful for distinguishing the events that occur in the proximal and distal intestine.

The protein-free diet failed to increase GLP-1 concentrations both in the PV and ILMV, indicating that protein is an essential component for the induction of GLP-1 release in response to the “meal”. This is partially supported by previous studies demonstrating that dietary proteins had a potent effect on GLP-1 secretion (20–23) as compared to that of carbohydrate and fat. As expected, profiles of PYY response were largely similar to that of GLP-1 responses in both experiments. This is consistent with the fact that these hormones are co-produced in L cells of the distal intestine, and with previous studies demonstrating the potent effect of dietary proteins on PYY secretion (21, 23, 24).

GLP-1 and PYY-producing cells exist abundantly in the ileum and colon, nevertheless these responses appeared within 15 min postprandially. Such quick responses might be mediated by neurohumoral reflexes triggered by sensing nutrients in the proximal small intestine (17–19, 25, 26).

Dietary fat is known to stimulate PYY in animal models and humans (2). Although all of test diet contained soybean oil at 7% (wt/wt), PYY concentrations did not increase in the ILMV after Pro-free diet ingestion (**Figure 3H**). In previous rat (approx. 300 g body weight) experiments, ileal infusion of 100 mM (28.2 mg/mL, 200 µL/min) oleic acid for 30 min (totally 169 mg oleic acid) induced much smaller PYY response than that of 5% peptone (totally 300 mg peptone) (27), and duodenal administration of 3 ml of 150 mM (42.3 mg/mL) oleic acid (totally 127 mg oleic acid = 1.14 kcal) induced much smaller PYY response than that of a liquid meal (totally 21 kJ = 5 kcal) (28). In the present study, rats ingested approximately 200 mg (70 mg fat/g diet x 2.5-2.8 g diet) of fat in MTTs. Because fat content was apparently lower than carbohydrate and protein contents in the diets, and these nutrients need to be emptied from the stomach into the small intestine to exert stimulatory effect on GIP/GLP-1/PYY secretions, we speculate that the amount of fat presented in the small intestine was not sufficient to trigger PYY secretion.

Insulin is not secreted from the intestine, and its secretion is stimulated by multiple factors such as GLP-1, GIP, glucose, amino acids, and fatty acid in the plasma, therefore, we are not able to provide relevant reasons for similar insulin responses observed in the present study regardless of the regions of blood sampling and of the diets given (**Supplementary Figure 1**).

Concentrations of plasma gut hormones could be affected by the rate of blood flow and by merging with the blood outside from the intestine such as the pancreas and kidney (29). Portal blood flow is increased by meal ingestion and plasma glucose (30–33). Decrements in GLP-1 concentrations observed in PV of rats fed the protein free diet may be due to dilution by these physiological factors. Blood sampling from a mesenteric vein tributary from the proximal small intestine would be desirable to exclude these effects, though it is technically challenging for repeated blood sampling in awake rats.

Liberated peptides (10, 11, 34–36) and/or amino acids (37, 38) from the dietary proteins likely stimulated GLP-1 release. We speculate that dipeptidyl peptidase-IV (DPP-IV) inhibitory peptides liberated from the dietary proteins (10, 34, 39–43) are absorbed in the small intestine, wherein they protect GLP-1 from degradation by DPP-IV in the plasma. Measurements of active GLP-1, total GLP-1 concentrations, their ratio, and DPP-IV activity in systemic bloods collected without full protease protection will be needed to examine the hypothesis in future studies. A previous study demonstrated greater effect of carbohydrates than proteins on GLP-1 release in conscious rats (44) using lymph fistula and duodenal nutrient infusion. The opposite conclusion to our study would be attributed to the differential experimental conditions.

It seemed surprising that the protein-free diet did not increase GLP-1 concentrations even though the diet mainly (more than 80% wt/wt) consisted of digestible carbohydrates. This could be because carbohydrates were rapidly digested and absorbed as monosaccharides in the proximal small intestine, as suggested by the data obtained for glycemic and GIP responses (experiment 1). The dose of the test diet at 10 g/kg was possibly insufficient to detect an elevation of GLP-1 concentrations for the protein-free (high carbohydrate) diet, even in the mesenteric and the portal veins. Indeed, we could not detect any increment in GLP-1 levels by oral administration of glucose or dextrin at the dose of 2-3 g/kg (45, 46), whereas oral administration of protein hydrolysates at 2 g/kg sufficiently enhanced GLP-1 concentrations in the peripheral vein of normal rats (10, 11).

It is unclear why the values of active GLP-1 concentration were higher than that of total GLP-1 concentration in the present study. The discrepancy might come from the difference in methodology (a multiplex assay system and a conventional ELISA kit). However, the postprandial responses were almost parallel between both forms of GLP-1s. Therefore, our results seem to appropriately represent GLP-1 secretory response at the postprandial state. Another limitation in the present study is that it is not possible to separate circulated blood from the ILMV and PV blood samples. In addition, comparing with postprandial GLP-1 concentrations in systemic blood samples would help to distinguish secreted, circulated, and degraded GLP-1s. If we can successfully cannulate into the cecal vein or mesenteric artery, it will be possible to evaluate the region-specific hormone responses or nutrient/drug absorption.

In summary, using novel ILMV cannulated model rats and comparing them to PV cannulated model rats, it was demonstrated that postprandial GLP-1 is released from the distal intestine under physiological conditions. Furthermore, by comparing postprandial GLP-1 responses to the diets with or without protein, it was revealed that protein in the meal has a critical role in triggering postprandial GLP-1 responses from the distal intestine.

## Data Availability Statement

The raw data supporting the conclusions of this article will be made available by the authors, without undue reservation.

## Ethics Statement

The animal study was reviewed and approved by The Hokkaido University Animal Committee.

## Author Contributions

TH, MS, and HH designed research. MS, and TH conducted research and analyzed data. TH and MS wrote the paper. TH had primary responsibility for final content. All authors contributed to the article and approved the submitted version.

## Funding

This work was supported by JSPS KAKENHI Grant Number 26252016 and 18K19158.

## Conflict of Interest

The authors declare that the research was conducted in the absence of any commercial or financial relationships that could be construed as a potential conflict of interest.
