# Supplementary material for: Blood Sampling From Rat Ileal Mesenteric Vein Revealed a Major Role of Dietary Protein in Meal-Induced GLP-1 Response
Source: Front Endocrinol (Lausanne). 2021 Jun 2;12:689685. doi: 10.3389/fendo.2021.689685 (PMC8206781; doi:10.3389/fendo.2021.689685)
Supplement: Supplementary file 1 [file DataSheet_1.docx]

**Supplementary Figure** **1.**

Insulin concentrations (A) and the AUC of insulin (B) in the portal vein (PV) plasma and in the ileal mesenteric vein (ILMV) plasma after ingestion of the standard diet, in Experiment 1, and changes (∆) in insulin concentrations in the PV plasma (C) and the ILMV plasma (D), and their AUC (E) after the ingestion of the diet-containing protein (control) or the diet-not containing protein (pro-free), respectively.

**Supplementary Figure** **2.**

Incremental area under the curve (∆AUC) of changes in glucose (A), active GLP-1 (B), total GLP-1 (C), total GIP (D), and PYY (E) concentrations summarized from the data in Experiment 2. P values shown in each panel were calculated by Student’s t-test to compare values between control and pro-free groups.
